# eCross-cultural adaptation of the spine oncology-specific SOSGOQ2.0 questionnaire to German language and the assessment of its validity and reliability in the clinical setting

**DOI:** 10.1186/s12885-021-08578-x

**Published:** 2021-09-23

**Authors:** T. Datzmann, W. Kisel, J. Kramer, M. Dreimann, J. D. Müller-Broich, C. Netzer, K. D. Schaser, J. Schmitt, A. C. Disch, the Tumor Study Group, Spine Section of the German Society of Orthopaedic and Trauma Surgeons (DGOU), K. D. Schaser, A. C. Disch, M. Dreimann, J. D. Müller-Broich, C. Netzer, D. Sauer, C. Heyde, R. Schmidt, M. Kreinest, M. Arand, U. Liljenqvist

**Affiliations:** 1 Center for Evidence-Based Healthcare, University Hospital Carl Gustav Carus, Technische Universität Dresden, Fetscherstraße 74, 01307 Dresden, Germany; 2 National Center for Tumor Diseases (NCT), Fetscherstraße 74, 01307 Dresden, Germany; 3 German Cancer Research Center (DKFZ), Im Neuenheimer Feld 280, 69120 Heidelberg, Germany; 4 Faculty of Medicine and University Hospital Carl Gustav Carus, Technische Universität Dresden, Fetscherstraße 74, 01307 Dresden, Germany; 5 Helmholtz-Zentrum Dresden - Rossendorf (HZDR), Bautzner Landstraße 400, 01328 Dresden, Germany; 6 University Comprehensive Spine Center (UCSC), University Center for Orthopedics, Traumatology and Plastic Surgery, University Hospital Carl Gustav Carus, Technische Universität Dresden, Fetscherstraße 74, 01307 Dresden, Germany; 7 Department of Trauma and Orthopedic Surgery, Center for Surgical Medicine, University Hospital Hamburg Eppendorf, Martinistraße 52, 20246 Hamburg, Germany; 8 Orthopedic University Hospital Friedrichsheim, Marienburgstraße 2, 60528 Frankfurt (Main), Germany; 9 Spine Surgery, University Hospital Basel, Spitalstrasse 21, 4031 Basel, Switzerland

**Keywords:** Cancer, Spinal malignancies, Health-related quality of life, Measurement comparison

## Abstract

**Background:**

The recently developed Spine Oncology Study Group Outcomes Questionnaire (SOSGOQ2.0) was proven a valid and reliable instrument measuring health-related quality of life (HRQOL) for patients with spinal malignancies. A German version was not available.

**Objective:**

A cross-cultural adaptation of the SOSGOQ2.0 to the German language and its multicenter evaluation.

**Methods:**

In a multistep process, a cross-cultural adaptation of the SOSGOQ2.0 was conducted. Subsequently, a multicenter, prospective observational cohort study was initiated to assess the reliability and validity of the German adaptation. To assess external construct validity of the cross-cultural adapted questionnaire, a comparison to the established questionnaire QLQ-C30 from the European Organisation for Research and Treatment of Cancer was conducted. Mean-difference plots were used to measure the agreement between the questionnaires in total score and by domain (deviation from mean up to 10% allowed). Further reliability and validity tests were carried out. Change to baseline was analysed 3–16 weeks later after different interventions occurred. Clinically relevant thresholds in comparison to the EORTC QLQ-C30 questionnaire were evaluated by ROC curve analysis.

**Results:**

We could enroll 113 patients from four different university hospitals (58 females, 55 males). Mean age was 64.11 years (sd 11.9). 80 patients had an ECOG performance status of 2 or higher at baseline. External construct validity in comparison to the EORTC QLQ-C30 questionnaire in total score and by domain was confirmed (range of deviation 4.4 to 9.0%). Good responsiveness for the domains *Physical Functioning* (*P* < .001) and *Pain* (*P* < .001) could be shown. The group mean values also displayed a difference in the domains of *Social Functioning* (*P* = .331) and *Mental Health* (*P* = .130), but not significant. The minimum clinically relevant threshold values for the questionnaire ranged from 4.0 to 7.5 points.

**Conclusions:**

According to our results, the cross-cultural adapted questionnaire is a reliable and valid tool to measure HRQOL in German speaking patients with spinal malignancies. Especially the domains *Physical Functioning* and *Pain* showed overall good psychometric characteristics. In this way, a generic questionnaire, such as the EORTC QLQ-C30, can be usefully supplemented by spine-specific questions to increase the overall accuracy measuring HRQOL in patients with spinal malignancies.

**Supplementary Information:**

The online version contains supplementary material available at 10.1186/s12885-021-08578-x.

## Introduction

The total number of patients with malignant spinal tumors increases continuously. With a constant number of new primary tumor cases, a significant increment in incidence of spinal metastases can be observed. Based on the growing success of adjuvant therapies with better and longer disease control [1] as well as the increased overall survival of the population and the associated risk of developing a malignant tumor the likelihood of spinal metastases equally raises. In all patients, whether in the rare cases of curative therapy approaches, but also in advanced tumor stages and limited treatment options, health-related quality of life (HRQOL), especially during and after extensive surgical interventions, is becoming an increasingly important monitoring-tool and target of therapy. HRQOL is not to be regarded as a symptom, but as interplay of several factors. To discriminate against these factors, there are different questionnaires from different professional societies, which record different dimensions of HRQOL [2]. The SF-36 [3, 4] or the WHOQOL questionnaire [5] are among the most important generic questionnaires. Tumor-specific questionnaires include the Functional Assessment of Cancer Therapy Questionnaire (FACT-G) [6] or the Rotterdam Symptom Checklist (RSCL) [7, 8]. The European Organisation for Research and Treatment of Cancer Quality of Life Questionnaire, EORTC QLQ-C30 [9], has also been developed for use in all cancer patients. With a remarkable majority of patients in palliative care the need arises to broaden the focus of treatment. Next to detailed clinical and laboratory parameters, more subjective, patient-centered outcomes were needed. Generic HRQOL measures were already established in the oncological setting at the hospital. The EORTC QLQ-BM22 [10] and FACT-BP questionnaires [11, 12] were available in German for quality of life studies in patients with bone lesions. However, little attention has been paid to the quality of life of patients with spinal metastases. A specific questionnaire to assess HRQOL of patients with malign tumors of the spine together with a generic questionnaire would increase the sensitivity and specificity of the assessment [13]. For this purpose, the Spine Oncology Study Group Outcomes Questionnaire (SOSGOQ) was developed, which showed excellent results regarding face and content validity [14] and proved to be a valid and reliable instrument in the clinical setting in English-speaking countries [14] as well as its revised second version SOSGOQ2.0 [13]. The questionnaire covers the dimensions of physical function, pain, mental health, social function and neurological function of the legs, arms, as well as the bowel and bladder on a 5-step Likert scale [13, 14]. Due to the lack of a German-language translation, this questionnaire could not be used in German-speaking countries.

## Methods

### Study design

**Fig. 1:**
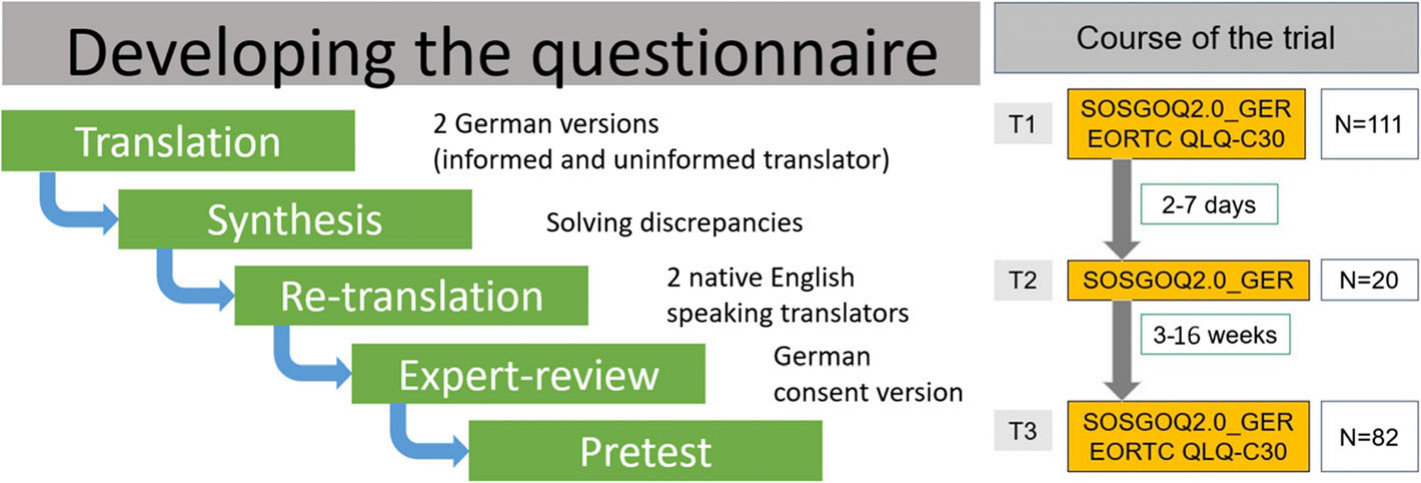
Schematic illustration of (left) the cross-cultural adaptation of the SOSGOQ2.0 questionnaire adapted after Beaton et al. 2000 and (right) the course of the trial with patient numbers which were used in the analyses

To achieve access to a disease-specific HRQOL questionnaire we decided to translate and culturally adapt the Spine Oncology Study Group Outcomes Questionnaire 2.0 (SOSGOQ2.0) from AOSpine International. After consent of the Knowledge Forum (KF) Tumor of the AOSpine an cross-cultural adaptation of the SOSGOQ2.0 was performed according to the published guidelines [15]. In a multistep translation and re-translation process, involving two native English speakers among others, the SOSGOQ2.0_GER was developed (Fig. 1, Table A1).

In January 2019 a multicenter, prospective observational cohort study was initiated to evaluate the reliability and validity of the cross-cultural adapted SOSGOQ2.0 questionnaire. Patients from three German centers and one from Switzerland were included after their written informed consent. Patients aged 18 years or older with a spinal malignancy were eligible for inclusion. Furthermore, they should be able to understand the German language and to answer the questionnaires independently. Ethics boards of each of the four participating hospitals approved the protocol (EK33012019, EK19–482, MC323/19, and EKNZ2020–00367). Information on demography, medical history, diagnostic procedures and findings, therapies including adverse events, and HRQOL data were gathered in a prospective manner. The time between baseline assessment (T1) and follow-up (T3) was 3–16 weeks. At least one intervention was performed in most patients during the interim period. Time point T2 after 2–7 days was used for reliability testing of the SOSGOQ2.0_GER and was only filled in by the first 20 patients. No intervention occurred in between and patients were asked at the same day time at both surveys. A schematic overview of the trial process is given on the right side of Fig. 1. After informed consent of the participants, the entire course of the survey was tested on 20 patients in a pre-test. This test was carried out to identify potential hurdles in the basic process as well as to test the comprehensibility of the questions and the handling of the questionnaires by the patients. Since there were no obvious discrepancies, we decided to include the data of these 20 pre-test patients into the final study.

### Statistical analyses

#### Reliability

The reproducibility of the individual answers was tested with a two one-sided t-test (TOST) [16] on the raw values of the SOSGOQ2.0_GER with an allowed discrepancy of 5 points (epsilon) and an alpha error of 10%. Equivalence was tested with the help of a specific package [17] for the statistical software R [18]. The same 20 patients were interviewed twice at time points T1 and T2, 2–7 days apart. Further, Cronbach alpha [19] among others was used to assess internal consistency of the domains.

#### Construct validity and case number calculation (primary outcome)

The primary outcome of this study was to validate our questionnaire results externally. Therefore, we also had all participants fill in the revised version 3.0 of the EORTC QLQ-C30 questionnaire in German language next to the SOSGOQ2.0_GER. The order was changed randomly. The structure of the SOSGOQ2.0_GER consists of five domains (Physical-, Neurological-, Social Functioning, Pain, and Mental Health). Since our main outcome was to evaluate the external (concurrent) construct validity of the SOSGOQ2.0_GER compared to a “gold standard” in oncological HRQOL assessment, like the EORTC QLQ-C30, we assessed only four domains which were also present in the standard. Questions about Neurological Functioning are absent in the EORTC QLQ-C30, but these are also not necessary to calculate the total score in the SOSGOQ2.0 questionnaire. Therefore, we excluded the questions 7–10 concerning neurological functions as well as post-therapy questions 21–27 in further analyses (Additional file 4).

Domains that are conceptually related were expected to be in agreement with each other. Mean-difference plots were used to assess the agreement of both questionnaire instruments. In both questionnaires HRQOL is measured on a point scale (1 indicates lower HRQOL than 2, etc.), therefore it is an ordinal scale. The Bland-Altman method takes into account not only the average difference of the measured values, but also the dispersion of the differences of the individual pairs of measured values and is particularly suitable for this comparison. It is a graphical procedure for assessing the agreement between two measurement methods [20]. Assuming a normal distribution of the errors, the limits of agreement can be calculated. Since both questionnaires have point scales between 0 and 100, a direct comparison was possible. Assuming a power of 80%, with 5% significance level and a permitted deviation of 10% between the measurement methods, a minimum case number of 86 patients was determined in advance [21, 22].

The internal structure of the SOSGOQ2.0_GER was evaluated by comparison of the item correlation within each domain and the correlation to items of other domains. If (a) the range of the correlation coefficients did not overlap and (b) the correlation within the domain was stronger than to any other domain, we counted this as an indication for the internal validity of the construct. We stratified into three groups, patients with (a) surgery and maybe other therapies, (b) patients with systemic- or radiotherapy exclusively, and (c) all patients together. We calculated Pearson correlation coefficients for baseline (T1) and follow-up (T3) data, separately. Numbers of patients within each group differ between the time points, since we used previous therapies before T1 for the assignment to groups in the baseline assessment and interventions between T1 and T3 for the assignment to groups in the follow-up analysis.

#### Clinical validity

We tested the SOSGOQ2.0_GER for its ability to differentiate between patient groups. Patients with an Eastern Cooperative Oncology Group (ECOG) performance score of 0 or 1 were compared to patients with an ECOG score ≥ 2. To measure response sensitivity in the clinical context, the course of disease (stable/improved vs. deteriorated) between T1 and T3 (within 3–16 weeks) was associated with changes in HRQOL scores.

#### Responsiveness to change and minimum clinically relevant change

The EORTC QLQ-C30 questionnaire is used as an external standard for testing response sensitivity. A clinically relevant change in this instrument indicates a change in the patient’s HRQOL, which should also be detected by the SOSGOQ2.0_GER (improvement/deterioration or stable disease). ROC curve analyses (sensitivity, specificity) were used to determine a threshold value with the highest quality of response sensitivity of the SOSGOQ2.0_GER questionnaire compared to the EORTC QLQ-C30 [23, 24]. The optimal threshold was determined domain by domain in the SOSGOQ2.0_GER compared to a fixed minimum clinically relevant change in the EORTC QLQ-C30 (> 5 points = change). The best model (“optimal threshold”) was chosen by optimizing sensitivity and specificity (accuracy) and then by ranking the results according to the highest positive predictive value (ppv). The chosen value indicates the best threshold compared to the EORTC QLQ-C30. A change between 5 and 10 points is indicated by the authors of the EORTC QLQ-C30 [9] as the minimum relevant change. Only more than 10 points are considered a moderate change.

In a further analysis, the change in HRQOL (measured by the EORTC QLQ-C30) was associated with change in the HRQOL scores of the SOSGOQ2.0_GER questionnaire. A Welch t-test was used to test of difference in means (level of significance: 0.05%). Statistical analyses were performed with the software R [18].

## Results

A total of 113 patients from three centers in Germany and one center from Switzerland were enrolled in a prospective observational cohort study from January 2019 until May 2020. The prostate (18%) was the most common primary tumor site, followed by the breast (13%) and multiple myelomas (10%). Baseline characteristics (T1) of the study population are shown in Table 1. At follow-up appointment 3–16 weeks (mean: 40 days; sd: 18.3 days) later (T3), complete data from 82 patients were remaining available for further analysis. Nine patients have died within this time period before they could be interviewed a second time. Two patients already had to be excluded from T1 as their data were incomplete and therefore the total scores of both questionnaires could not be calculated. Furthermore, 20 patients had to be excluded to T3 for the same reason. Most patients (85) were hospitalised at the time of baseline assessment. 83 patients received prior to study inclusion a surgical treatment, 46 patients received radiotherapy, and 50 patients received a systemic therapy. Between T1 and T3, 25 patients underwent surgical treatment, 41 patients received radiotherapy, 23 patients received a systemic therapy, 19 patients received a different therapy and 26 patients received no therapy at all. These categories may overlap.

**Table 1:** Baseline characteristics of the study population

Characteristic	No. (%)
Number of patients	113 (100)
Mean age at inclusion	64.1 (SD 11.9)
Sex	
Female	58 (51.3)
Male	55 (48.7)
ECOG Score	
0–1	32 (28.3)
2–4	80 (70.8)
Unclear	1 (0.9)
Primary tumor	
Prostate Cancer	20 (17.7)
Breast Cancer	15 (13.3)
Multiple Myeloma	11 (9.7)
Renal Cell Cancer	10 (8.8)
Lung Cancer	9 (8.0)
Others	48 (42.5)
Treatment intention	
Palliative	80 (70.8)
Curative	15 (13.3)
Unclear	18 (15.9)
Previous treatment (categories may overlap)	
Surgery	83 (73.5)
Systemic therapy	50 (44.2)
Radiotherapy	46 (40.7)

*Abbreviations*: *ECOG* Eastern Cooperative Oncology Group performance status, *SD* Standard Deviation

### Reliability of the measurement results

The retest was filled in by the first 20 patients from one center within 2–7 days after the baseline assessment. No intervention took place in between. The TOST test on the raw HRQOL scores revealed no significant difference within the given confidence limits (*P* < .001). Thus the SOSGOQ2.0_GER questionnaire is a reliable measurement instrument, which is the basic requirement for its application.

Cronbachs alpha was mainly used to evaluate internal consistency of the domains of the SOSGOQ2.0_GER. In order to better understand the values of the SOSGOQ2.0_GER, the values for the EORTC QLQ-C30 questionnaires were given for comparison. All questions in the domains *Physical Functioning* and *Pain* got high Cronbach alpha values above 0.7 in both questionnaires with one exception in the domain *Pain* for EORTC QLQ-C30 (Table 2). The domains *Mental Health* and *Social Functioning*, however, showed significantly lower values. For the domain *Mental Health* this also applies to the EORTC QLQ-C30, but in *Social Functioning* only the SOSGOQ2.0_GER showed very low Cronbach alpha values.

**Table 2:** Internal Consistency of the SOSGOQ2.0_GER in comparison to the EORTC QLQ-C30 domains measured at baseline assessment. Cronbach alpha values below 0.70 are a sign of poor consistency

SOSGOQ2.0_GER					EORTC QLQ-C30			
Domains	Questions	Cronbachs alpha	average interitem correlation	item correlation with domain score	Questions	Cronbachs alpha	average interitem correlation	item correlation with domain score
***Physical functioning***	Overall	0.90	0.59	–	Overall	0.90	0.66	–
	1) activity	0.88	0.59	0.81	1)	0.89	0.67	0.82
	2) employability	0.87	0.57	0.86	2)	0.88	0.65	0.86
	3) independence	0.87	0.57	0.86	3)	0.86	0.63	0.91
	4) agility	0.87	0.57	0.88	4)	0.87	0.64	0.88
	5) walking	0.89	0.63	0.74	5)	0.90	0.71	0.80
	6) social life	0.89	0.63	0.74				
***Pain***	Overall	0.88	0.59	–	Overall	0.88	0.78	–
	11) intensity	0.85	0.6	0.8	9)	0.78	0.78	0.94
	12) persistency	0.84	0.58	0.84	19)	0.62	NA	0.95
	13) mobility	0.83	0.55	0.87				
	14) coping	0.84	0.57	0.84				
	15) overwhelming	0.87	0.64	0.75				
***Mental health / Cognitive Functioning***	Overall	0.53	0.36	–	Overall	0.48	0.32	–
	16) depression	0.36	0.36	0.81	20)	0.32	0.32	0.86
	17) anxiety	0.13	NA	0.84	25)	0.1	NA	0.76
***Social Functioning***	Overall	0.52	0.25	–	Overall	0.8	0.67	–
	18) concentration	0.31	0.19	0.77	26)	0.67	0.67	0.92
	19) relationships	0.18	0.1	0.81	27)	0.45	NA	0.91
	20) new people	0.63	0.46	0.54				

### Clinical validity

ECOG data was available for 112 patients at baseline, 80 of which had a performance status of 2 or higher. Good differentiation of the SOSGOQ2.0_GER sum score between patients with low (0 or 1) and high ECOG (≥2) scores at baseline was achieved (*P* < .001, Welch t-test). Table 3 shows the responsiveness to change in the domains of the SOSGOQ2.0_GER within the course of the disease. Patients with a stable or improved condition (*N* = 71) had an increase in domain scores for *Pain* and *Mental Health* indicating an improvement of their HRQOL. However, in the domain *Physical Functioning* a slightly deteriorated score (Mean in change − 0.6) was detected. But compared to the patients with deterioration of their condition (*N* = 10), the worsening of the *Pain* scores (Mean in change − 4.6) was not as severe. These patients showed a decrease in the scores also in the *Social Functioning* domain (Mean in change − 1.6), but much weaker than the patients with deterioration of disease. Unfortunately, because of low case numbers within one group, statistical test of difference in means (t-test), were not significant.

**Table 3:** Response Sensitivity of the SOSGOQ2.0_GER to the course of the disease

Change in Domain Mean (SD) MIN/MAX	Deterioration of Disease *N =* 10	Disease Stable/Improved *N =* 71	Total *N* = 82	*P*
Physical Functioning	−4.6 (33.3) −46/59	−0.6(21.4) −63/71	−1.1 (22.8) −63/71	0.613
Pain	5.0 (33.2) −55/50	13.4 (25.7) −35/85	12.2 (26.5) −55/85	0.355
Mental Health	−1.2 (35.8) −50/63	4.2 (25.3) −38/100	3.6 (26.4) −50/100	0.551
Social Functioning	−9.3 (22.8) −50/16	− 1.6(20.2) −50/42	−2.4 (20.4) −50/42	0.267

*Abbreviation*: *SD* Standard Deviation

### External construct validity (primary outcome)

**Fig. 2:**
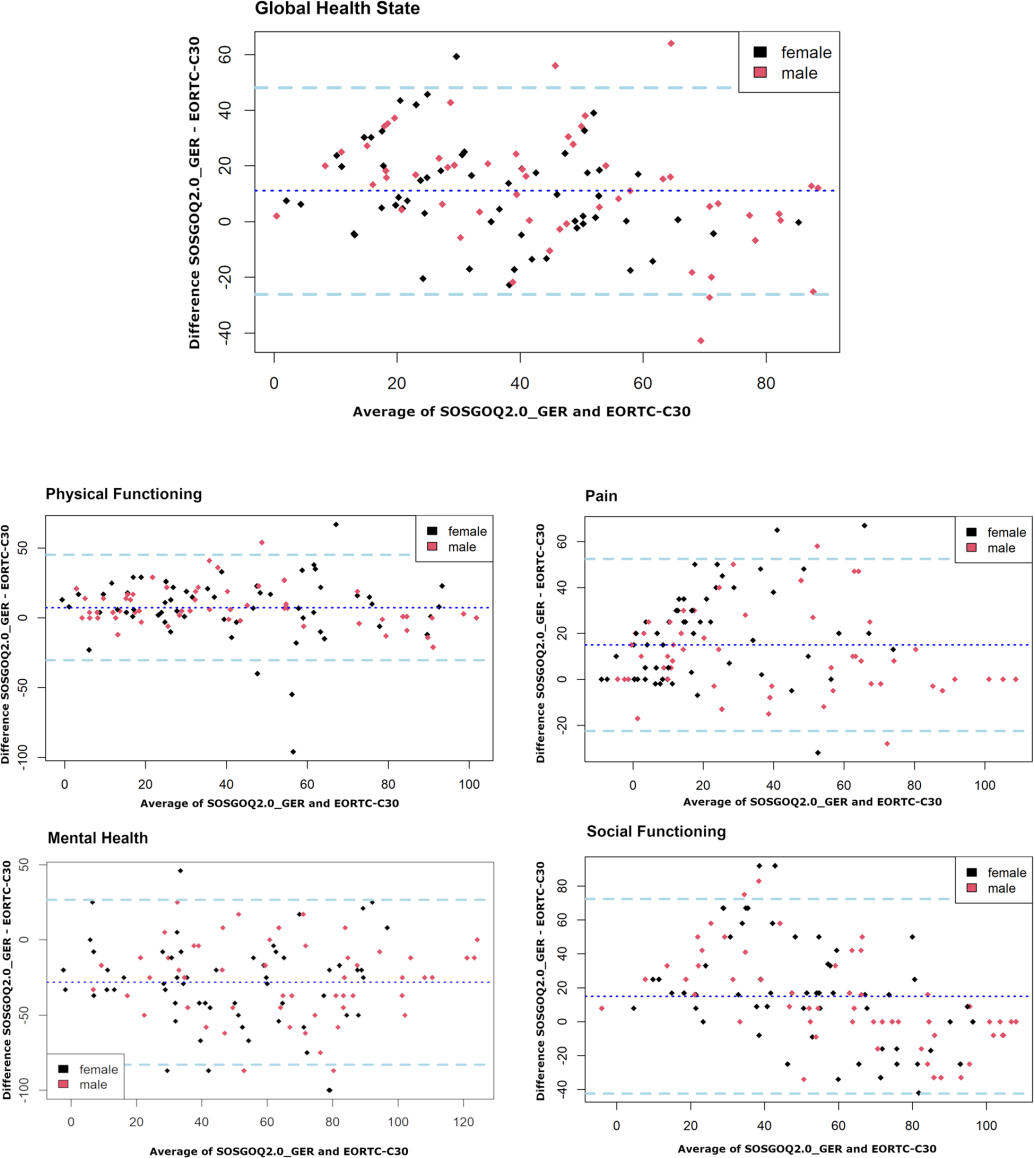
Above: Mean-Difference Plot for the comparison of the Global Health State measured by the SOSGOQ2.0_GER and the EORTC QLQ-C30 questionnaires at baseline assessment. The thick dotted lines at the top and bottom of the figure represent the limits of agreement. Compared are 111 patients where the x-axis shows the average versus the difference of both measurements on the y-axis. Six of 111 comparisons are outside or intersect with the limits of agreement corresponding to an error slightly higher than 0.05. By chance alone we would expect 5 % background noise under the assumption that the error is normally distributed. But in advance (see material and methods) we have determined that we will tolerate a disagreement of 10 % between the measurement methods. Women and men are color-coded for representation purposes only. Below: Mean-Difference Plots for the comparison domain by domain at baseline assessment. Domain (number of patients, disagreement in percent) - Physical Functioning (113, 4.4), Pain (112, 4.5), Mental Health (111, 9.0), Social Functioning (111, 4.5). All domains were within the allowed error of 10 %

To evaluate validity of the SOSGOQ2.0_GER we compared it to the EORTC QLQ-C30, a valid and reliable generic cancer-specific questionnaire which is used for patient-reported HRQOL assessments. Data on a total of 113 patients were available. Of these 111 could be used for the comparison of the measurement methods at baseline. Bland-Altman method provided excellent agreement between the total scores of both instruments (Fig. 2). The deviation was 5.4%. The good agreement between the two questionnaires could also be confirmed separately for each domain (*Physical Functioning*, *Pain*, *Mental Health*, and *Social Functioning*). All deviations were within the allowed range (Fig. 2). The construct “health-related quality of life” is therefore measured comparably by both instruments.

### Evaluation of the internal structure

The internal structure of the SOSGOQ2.0_GER was evaluated by correlating items with its own domain and with the items of the other domains (Table 4). The patients were evaluated in 3 groups: (a) with surgery ± systemic therapy/radiotherapy (CTx/RTx), (b) with CTx/RTx only, and (c) all patients together. The first white line in Table 4 always indicates values from the baseline assessment, while grey lines show values from the follow-up after 3–16 weeks. Number of patients differs between time points, since previous therapies before T1 were used for allocation in the baseline assessment, while therapeutical interventions between T1 and T3 were used for allocation to the groups in the follow-up. The correlations with the own domain were always much higher and, with a few exceptions, there was no overlap in the ranges of the correlation coefficients (exceptions are printed in bold). The domains *Physical Functioning*, *Pain* and *Mental Health* were robust, with one outlier in the domain *Physical Functioning* in the follow-up assessment in the group with a surgical intervention between T1 and T3 and with another outlier in the domain *Mental Health* in the baseline assessment in the group with CTx/RTx exclusively. But here the case numbers were very low with 20 patients respectively 35 patients, which makes it difficult to achieve statistical significance. However, in the domain *Social Functioning* there were overlaps in all studied groups. Especially in the follow-up assessment all correlations were overlapping. The case numbers here ranged from 20 to 82 patients. With the exception of the *Social Functioning* domain, our estimates support the internal validity of the SOSGOQ2.0_GER domains.

**Table 4:** Convergent and Divergent Validity at baseline and at 3–16 weeks after treatment

Domains	Surgery ±CTx/RTx		N	CTx/RTx Only		N	All Patients		N
	Item Own Domain Correlation	Item Other Domain Correlation		Item Own Domain Correlation	Item Other Domain Correlation		Item Own Domain Correlation	Item Other Domain Correlation	
Physical Functioning	0.71–0.88	0.24–0.38	83	0.78–0.90	0.40–0.62	35	0.74–0.88	0.27–0.46	113
	**0.39–0.87**	**0.51–0.64**	20	0.65–0.90	0.53–0.61	41	0.75–0.92	0.58–0.64	82
Pain	0.70–0.83	0.37–0.58	83	0.82–0.89	0.51–0.69	35	0.75–0.87	0.45–0.58	113
	0.65–0.86	0.51–0.58	20	0.75–0.87	0.56–0.70	41	0.75–0.87	0.54–0.58	82
Mental Health / Cognitive Functioning	0.79–0.90	0.35–0.58	83	**0.75–0.79**	**0.58–0.77**	35	0.81–0.84	0.42–0.58	113
	0.78–0.84	0.58–0.64	20	0.88–0.89	0.53–0.70	41	0.87–0.88	0.58–0.67	82
Social Functioning	0.55–0.80	0.24–0.49	83	**0.51–0.87**	**0.40–0.77**	35	0.54–0.81	0.27–0.47	113
	**0.58–0.92**	**0.53–0.64**	20	**0.49–0.83**	**0.61–0.65**	41	**0.53–0.83**	**0.54–0.67**	82

*Abbreviations*: *CTx* chemotherapy, *RTx* radiotherapy, first line shows the values from the baseline survey (T1), second line with gray background shows the values for the follow-up (T3); values printed in bold mark overlaps between the domains; N number of patients

### Sensitivity to change

Table 5 shows response sensitivity of the SOSGOQ2.0_GER compared to the EORTC QLQ-C30 questionnaire. The determined minimum clinically-relevant thresholds in SOSOGOQ2.0_GER vary between 4 and 7.5 points, depending on the domain. All domains reach high to acceptable sensitivities [19]. However, the specificities are significantly worse in the two domains *Mental Health* and *Social Functioning* compared to *Physical Functioning* and *Pain*, which is also reflected in the low positive predictive values of both domains. It is interesting to note that in the domains *Physical Functioning* and *Pain*, fewer patients in the SOSGOQ2.0_GER change in their HRQOL between T1 and T3 compared to the assessment with the EORTC QLQ-C30, while in the domains *Mental Health* and *Social Functioning* the opposite is true.

**Table 5:** Responsiveness of the SOSGOQ2.0_GER questionnaire to the EORTC QLQ-C30

***EORTC QLQ-C30***	***SOSGOQ2.0_GER***						
Patients with treatment-related change		Domain	thr	spe	sen	acc	ppv
65	51	Physical Functioning	6.5	0.71	0.71	0.71	0.90
59	52	Pain	7.5	0.65	0.75	0.72	0.85
53	61	Mental Health	6.0	0.31	0.77	0.61	0.67
61	71	Social Functioning	4.0	0.24	0.90	0.73	0.77

*Abbreviations*: *thr* threshold, *spe* specificity, *sen* sensitivity, *acc* accuracy, *ppv* positive predictive value; variation in the EORTC QLQ-C30 questionnaire of more than 5 points was considered as an actual change

In a further analysis the patients to T3 were stratified into one group with a stable or improved EORTC QLQ-C30 score and another group with deterioration (Table 6). Domain by domain the mean of the changes of the SOSOGOQ2.0_GER scores could now be compared between the groups and tested for differences. Patients with stable or improved condition (*N* = 57) showed positive mean values for the change in HRQOL, indicating an improvement within these patients. An exception is the domain *Social Functioning*, where a slight deterioration of the QOL scores (Mean in change − 0.9) could be seen. Patients with deterioration of disease (*N* = 25) showed mostly negative mean values in the SOSGOQ2.0_GER scores indicating the worsening of their condition. Here, the Pain domain score (Mean in change + 0.2) showed almost no change as the only exception. A significant difference in means could only be proven for the domains *Physical Functioning* and *Pain* (*P* < .001).

**Table 6:** Response Sensitivity of the SOSOGOQ2 based on change in the EORTC-C30

Change in Domain Mean (SD) MIN/MAX	C30 Decline *N* = 25	C30 Stable/ Improved *N* = 57	Total *N* = 82	*P*
Physical Functioning	−13.6 (24.2) −63/29	4.4(20.0) −62/71	−1.1(22.8) −63/71	< 0.001
Pain	0.2 (27.4) −55/65	17.5 (24.6) −35/85	12.2 (26.5) −55/85	< 0.001
Mental Health	−3.0 (31.2) − 50/88	6.6 (23.8) −38/100	3.6 (26.4) − 50/100	0.130
Social Functioning	−5.7 (22.3) −50/41	−0.9(19.5) − 50/42	−2.4 (20.4) − 50/42	0.331

Overall, the domains *Pain* and *Mental Health* indicated an improvement in HRQOL on average of the total cohort after 3–16 weeks. While the cohort in the domains *Physical Functioning* and *Social Functioning* slightly deteriorated on average at the same time.

## Discussion

Patients in high tumor stages with bone metastasis and the associated restrictions in terms of resilience, mobility and pain represent a challenge in assessing HRQOL. Especially here, the use of a disease-specific questionnaire is recommended in addition to generic instruments for measurement of HRQOL. Since there was no specific German questionnaire for patients with spinal malignancies, we aimed - following consent given by the AOKnowledge Forum Tumor - to cross-cultural adapt the Spine Oncology Study Group Outcomes Questionnaire (SOSGOQ2.0) and test it clinically. While primary spinal tumors are an absolute rarity, spinal metastases show a 250-fold higher prevalence. According to a study in the US, the cumulative incidence of bone metastases among solid tumors was 2.9% after 30 days, 4.8% after 1 year, 5.6% after 2 years, and 9% after 5 years. This varies by cancer type, with patients suffering from prostate cancer showing the highest risk at 18–29%, followed by lung, kidney, and breast cancer. In patients with tumors of stage IV malignancy at the time of initial diagnosis, the cumulative incidence after 30 days was as high as 11% [25]. Although we cannot calculate comparable numbers in our setting, prostate cancer, followed by breast, kidney and lung cancer were also among the most common primary tumors our patients. Therefore it seems to reflect the common heterogeneity of the spinal metastases cohort. However, the cross-cultural adapted questionnaire SOSGOQ2.0_GER equally displayed the different domains independent from the entity.

### Psychometric properties of the questionnaire

The evaluation of the adapted questionnaire (SOSGOQ2.0_GER) showed that it is a valid and reliable tool and therefore well suited as supplement to a generic questionnaire, like the EORTC QLQ-C30. By comparison of the SOSGOQ2.0_GER and the EORTC QLQ-C30 score we showed a high agreement between the measurement methods, which confirms the construct validity of the SOSGOQ2.0_GER externally. Sufficient test-retest reliability was confirmed for all four examined domains. The analyses of internal consistency showed excellent values (Cronbachs alpha, item correlation) for the two domains *Physical Functioning* and *Pain*. The domains *Mental Health* and *Social Functioning*, however, showed less consistency. To get closer to the bottom of this result we calculated the same consistency measures for the EORTC QLQ-C30 for our patients. In the EORTC QLQ-C30 questionnaire the domain *Mental Health* showed also low consistency values, but not in the domain of *Social Functioning*. This could indicate that our patients generally have a changing mental state that is strongly influenced by the severity of their symptoms and the accompanying treatments (e.g. systemic therapy). This and the small size (2 questions) of the *Mental Health* domain could explain the lower reliability measures. However, the lower values for the *Social Functioning* domain could have a further intrinsic reason. This domain consists of the three questions 18 to 20. If we take out question 20, the average inter-item-correlation is more than doubled, to almost acceptable values. Thus, question 20 seems a problem. Here we asked the patient if she/he feels comfortable meeting new people. Questions 18 and 19 of the same construct contain the specific reference that the influence due to the spinal cord should be addressed. This reference is missing in question 20. In addition, it is more common in Germany to ask about the feeling of discomfort and not about well-being when it comes to getting to know new situations. Therefore we suggest a correction of question 20: *“Does your spinal disease make you feel more uncomfortable when you meet new people?”* The scale has to be reversed, of course. You will find a German adaption to this in the supplement (Table A2).

Two domains, *Physical Functioning* and *Pain*, showed good responsiveness compared to changes in the domains of the EORTC QLQ-C30 questionnaire. This was indicated by accuracies over 70% and high positive predictive values. One reason for the poor performance (accuracies below 70%) of the domains *Social Functioning* and *Mental Health* could be the lower number of questions in these constructs. Another alternative is more general and concerns the very specific patient population of this study. It could already be shown, e.g. by Jocham et al. [26], that HRQOL measurements in patients in advanced tumor stages (often reflected by spinal metastases) do not always lead to valid and reliable results, especially in the area of *Mental Health* and *Social Functioning*. This is also indicated by the poor internal consistency of the domain *Mental Health* in the SOSGOQ2.0_GER as well as in the EORTC QLQ-C30 questionnaire within our patient cohort.

We were able to determine minimum clinically relevant threshold values for each domain by ROC curve analysis. The thresholds ranged between 4.0 and 7.5 points on a hundreds scale. This is in the range of the minimum clinically relevant threshold values for the EORTC QLQ-C30 questionnaire. Here the authors [9] have calculated 5–10 points.

### Clinical application

In the clinical application disease-specific monitoring of HRQOL should ideally display outcome of therapeutical approaches and enable the multidisciplinary oncological team to reflect the impact of the different valuable treatment options during the course of the malignant disease. But moreover, it has to be an operational tool to modify decisions and choose alternative treatment branches and even overall treatment strategies. For most primary spinal tumors – especially sarcomas - treatment strategy is a radical surgical resection combined with neo−/adjuvant therapies [27, 28]. Dea et al. showed in a meta-analysis that patients profit from so called “Enneking-appropriate” resections with increasing survival time from surgery in terms of HRQOL despite of the surgical complexity, associated risks and complication rates. In turn, a fail to reach resection goals inevitably leads to deterioration in the course of the malignant disease due to directly related higher local recurrence rates and decreased overall survival [29]. The authors concluded that wide resections are justified to elevate long-term patients` HRQOL and to maximize the outcome and they recommended treating these rare entities in specialized spine-oncological centers exclusively. While treatment algorithms for primary tumors are not doubtful, decision making for spinal metastases treatment is even more diverse. Neurological deficits due to metastatic invasion of the spinal canal directly impair physical function and thereby overall HRQOL. In the acute clinical situation emergency surgical intervention is indicated to protect sensory and motoric function and in ideal circumstances to allow further mobility even in palliative treatment situations. Tumors that impair spinal integrity can nowadays be classified by different scores (e.g. Spinal Instability Neoplastic Score – SINS), that are used as a guideline to judge about destabilizing factors of a lesion and the necessity to surgically stabilize the spine. Unstable classified spinal lesions present with an impaired outcome when solely irradiated and not surgically stabilized. In turn, radiation therapy alone is of high success in stable but painful lesions [30, 31] resulting in adequate quality of life. However, difficulties arise when patients present with so called “potentially unstable” lesions without neurological deterioration. Aside of the clinical and radiological constellation, disease-specific HRQOL tools could give a further aid to develop decisions. In an ideal situation, decisions are based on a full understanding, but this ideal is hard to achieve, particularly for malignant diseases. The problem of decision making in cancers is known to be compounded by a variety of psychological limitations of involved individuals (e.g. risk aversion, ambiguity aversion, etc.). Therefore the less reliable domains of social functioning and mental health in different questionnaires might also bias decisions. To overcome that problem a close integration of patients and their related persons (“shared” or “patient-centered” decision making - SDM) might be a solution and was demanded in different publications reviewed in Reyna et al. [32].

### Strengths and limitations

A particular strength of our study results from the multicenter approach. All four participating university hospitals meet high clinical standards. Therefore, we were able to achieve excellent documentation and high data quality, which is reflected in a high completeness of the questionnaire data. Furthermore, the multicenter approach made it possible to test the cross-cultural adapted SOSGOQ2.0_GER questionnaire on a broader spectrum of patients, considering different facets of the German language. In addition, the necessary number of cases could be achieved quickly. This limited the actual needed study duration.

As the required number of cases was calculated based on the primary endpoint, in some cases only low power could be achieved in analyses of secondary endpoints. Therefore, these derived statistics are not as reliable. Some of the stratified analyses struggle with a small number of cases.

Only short-term effects were analyzed, a maximum of 16 weeks after the intervention, but possible long-term effects on HRQOL were not considered.

## Conclusion

The SOSGOQ2.0_GER questionnaire is a reliable and valid instrument to measure HRQOL in patients with malignant spinal tumors. The domains of *Physical Functioning* and *Pain* showed good psychometric properties. The domains *Mental Health* and *Social Functioning* are represented by fewer questions and showed discrepancies in the consistency and response sensitivity analyses. Especially the domain *Social Functioning* showed poor internal consistency (e.g. low Cronbach alpha value). It was therefore necessary to adjust this domain and correct an imprecise formulation of a question. This proposed change still needs to be tested in a follow-up study. However, we can recommend using this spine-specific questionnaire to measure HRQOL in patients with malignant spinal tumors in addition to a generic questionnaire, such as the EORTC QLQ-C30.

## Supplementary Information

**Additional file 1: Table A1 -** Back translation of the German pre-final version into English language and final translated version of the SOSGOQ2.0_GER questionnaire

**Additional file 2: Table A2 -** Adaptation of question 20 into German language. Note that the answer categories remain unchanged, however, the scale must be reversed.

**Additional file 3: A3 - Supporting Information -** Scoring manual SOSGOQ2.0_GER (German version)

**Additional file 4:** Structure of the Spine Oncology Study Group Outcomes Questionnaire 2.0 GERMAN (SOSGOQ2.0_GER) culturally adapted to German speaking people. Questions 1–6 represent the domain Physical Functioning, 7–10 Neurological Functioning, 11–15 Pain, 16/17 Mental Health, 18–20 Social Functioning, and 21–27 are post-therapy questions. In the supplement you will find a comparison of the back translated questions into English language performed independently by two native speakers and the final German adaptions (Table A1) as well as a German scoring manual (Table A3).

## Data Availability

The primary data are subject to further analysis, but are available from the authors upon reasonable request.
