# Supplementary material for: eCross-cultural adaptation of the spine oncology-specific SOSGOQ2.0 questionnaire to German language and the assessment of its validity and reliability in the clinical setting
Source: BMC Cancer. 2021 Sep 23;21:1044. doi: 10.1186/s12885-021-08578-x (PMC8459467; doi:10.1186/s12885-021-08578-x)
Supplement: Supplementary file 1 — Additional file 1: Table A1 - Back translation of the German pre-final version into English language and final translated version of the SOSGOQ2.0_GER questionnaire [file 12885_2021_8578_MOESM1_ESM.docx]

**Table A1** – Back translation Into English language

| **Final Translated Version (T-12)** | **Back-Translated Version**  **Dr. Hegewald** | **Back-Translated Version Dr. Nail** |
| --- | --- | --- |
| *Title* |  |  |
| *Spine Oncology Study Group* Fragebogen zur Lebensqualität 2.0 (deutsche Version) | *Spine Oncology Study Group* Quality of Life Questionnaire 2.0 (German Version) |  |
| *Patient information* | *Patient information* |  |
| Patientenname | Patient name | Patient name |
| Datum (DD/MM/YY) | Date (MM/DD/YYYY) | Date (day/month/year) |
| Patientennummer (vom Klinikpersonal auszufüllen) | Patient number (to be filled out by clinic personnel) | patient number (to be filled out by administration) |
| *Instructions* | *Instructions* |  |
| Hinweise: In diesem Fragebogen werden Sie gebeten, Ihren Gesundheitszustand einzuschätzen. | Note: In this questionnaire, you will be asked to rate your health status. | In this questionnaire you will be asked to assess your health condition |
| Bitte betrachten Sie beim Beantworten der Fragen Ihre körperlichen Fähigkeiten und Symptome innerhalb der letzten 4 Wochen. | When answering the next questions, please consider your physical capacity and symptoms within the last 4 weeks. | Please assess your physical capabilities and symptoms within the last 4 weeks. |
| Es ist wichtig, dass Sie jede Frage selbst beantworten. | It is important that you answer question yourself. | It is important that you answer the questions yourself. |
| Bitte geben Sie pro Frage nur eine Antwort an. | Please give only one answer per question. | Please provide only one answer per question. |
| Die Fragen 21-27 sollten nur nach abgeschlossener Behandlung zu den Nachuntersuchungen beantwortet werden. | The questions 21-27 should only be answered after the treatment of the follow-up examinations is completed. | The questions 21 - 27 should only be answered after completion of the final examinations. |
| *Item* | *Item* |  |
| Vom Patienten auszufüllen | To be filled out by the patients | To be filled out by the patient |
| 1. Wie beurteilen Sie Ihre Leistungsfähigkeit? | 1. How do you judge your capabilities? | 1. How would you assess your physical capability? |
| - Alle Aktivitäten ohne Einschränkung - Moderate Aktivitäten außer Haus - Mobilität auf zu Hause beschränkt - Mobilität vom Bett zum Stuhl - Bettgebunden | - All activities without limitations - Moderate activities outdoors - Mobility limited to indoors - Mobility from bed to chair - Bedridden | All activities without exception      Moderate activities outside of the house      Mobile at home      Mobile from bed to chair      Bedridden |
| 1. Wie beurteilen Sie Ihre Arbeitsfähigkeit (Beruf / Haushalt)? | 1. How do you judge your ability to work (including household/studies)? | 1. How would you assess your work capabilities (including household/ study)? |
| - Uneingeschränkt - 4-8 Stunden am Tag - 2-4 Stunden am Tag - Weniger als 2 Stunden am Tag - Keine Arbeit möglich | - Unlimited / no limitations - 4-8 hours per day - 2-4 hours per day - Less than 2 hours per day - Unable to work | Unlimited      4-8 h per day      2-4 h per day      Work not possible |
| 1. Sind Sie aufgrund Ihrer Wirbelsäule in Ihrer Fähigkeit für sich selbst zu sorgen eingeschränkt? | 1. Are your abilities to care for yourself limited due to your spine? | 1. Are you limited in your daily activities due to your spine? |
| - Überhaupt nicht - Ein wenig - Etwas - Ziemlich - Sehr stark | - Not at all - A little - Somewhat - Rather - Extremely | Not at all      A bit      Somewhat      Quite a bit      Very much |
| 1. Benötigen Sie für Aktivitäten außerhalb Ihres Zuhauses Hilfe von anderen? | 1. Do you need help from others for activities outside of the house? | 1. Do you need help from others for your activities at home? |
| - Nie - Selten - Manchmal - Oft - Sehr oft | - Never - Seldom - Sometimes - Often - Very often | Never      Seldom      Sometimes      Often      Very often |
| 1. Welche Unterstützung benötigen Sie beim Gehen? | 1. What kind of assistance do you need for walking? | 1. Which help do you need for work? |
| - Keine - Eine Gehhilfe - Einen Rollator / 2 Gehhilfen - Hilfe anderer - Kein Gehen möglich | - None - A cane/ A crutch - A walker/ crutches - Help from others - Unable to walk | None     Crutch or walking stick      Wheel chair/ 2 crutches      Other help      Not possible to walk |
| 1. Verlassen Sie das Haus für soziale Aktivitäten? | 1. Do you leave the house for social activities? | 1. Do you leave the house for social activities? |
| - Nie - Selten - Manchmal - Oft - Sehr oft | - Never - Seldom - Sometimes - Often - Very often | Never      Seldom      Sometimes      Often      Very often |
| 1. Haben Sie eine Schwäche der Beine? | 1. Do you have a weakness in the legs? | 1. Do you have weakness in your legs? |
| - Keine - Gelegentlich leicht - Dauernd leicht - Dauernd mäßig - Dauernd stark | - None - Sometimes light - Always light - Always moderate - Always strong | Never      Sometimes-light      Always - light      Always - moderate      Always - severe |
| 1. Haben Sie eine Schwäche der Arme? | 1. Do you have a weakness in the arms? | 1. Do you have weakness in your arms? |
| - Keine - Gelegentlich leicht - Dauernd leicht - Dauernd mäßig - Dauernd stark | - None - Sometimes light - Always light - Always moderate - Always strong | Never      Sometimes-light      Always - light      Always - moderate      Always - severe |
| 1. Haben Sie Schwierigkeiten, Ihre Darmfunktion (außer bei Durchfall / Verstopfung) zu kontrollieren? | 1. Do you have difficulty controlling your bowel movements (except diarrhea/ constipation)? | 1. Do you have difficulties controlling your bowel movements (other than diarrhea/ constipation)? |
| - Nie - Selten - Manchmal - Oft - Sehr oft | - Never - Seldom - Sometimes - Often - Very often | Never      Seldom      Sometimes      Often      Very often |
| 1. Haben Sie Schwierigkeiten, Ihre Blasenfunktion zu kontrollieren? | 1. Do you have problems controlling your bladder function? | 1. Do you have difficulties controlling urination? |
| - Nie - Selten - Manchmal - Oft - Katheter erforderlich | - Never - Seldom - Sometimes - Often - Catheter required | Never      Seldom      Sometimes      Often      Catheter necessary |
| 1. In welchem Ausmaß haben Sie insgesamt Rücken-/Nackenschmerzen? | 1. To what degree do you have back/neck pain altogether? | 1. How intense is your back/neck pain in general? |
| - Keine - Sehr leichte - Leichte - Mäßige - Starke | - None - Very light - Light - Moderate - Strong | Not      Very light      Light      Moderate      Severe |
| 1. Wenn Sie sich in Ihrer bequemsten Körperposition befinden, haben Sie weiterhin Rücken-/Nackenschmerzen (welche Ihren Schlaf stören)? | 1. Do you have back/neck pain (which disturbs your sleep) when you are in your most comfortable body position? | 1. When you are in your most comfortable body position, do you still have back/neck pain (which disturb your sleep)? |
| - Nie - Selten - Manchmal - Oft - Sehr oft | - Never - Seldom - Sometimes - Often - Very often | Never      Seldom      Sometimes      Often      Very often |
| 1. Wie oft beeinträchtigen die Schmerzen Ihre Beweglichkeit (Sitzen, Stehen, Gehen)? | 1. How often does pain impair your mobility? | 1. How often does your pain limit your mobility (sitting, standing, walking)? |
| - Nie - Selten - Manchmal - Oft - Dauernd | - Never - Seldom - Sometimes - Often - Always | Never      Seldom      Sometimes      Often      Very often |
| 1. Wie sicher fühlen Sie sich in Ihren Möglichkeiten, Ihren Schmerz selbständig zu bewältigen? | 1. How certain do you feel with your possibilities to manage your own pain? | 1. How sure are you that you can manage your pain by your self? |
| - Überhaupt nicht sicher - Wenig sicher - Mäßig sicher - Meistens sicher - Völlig sicher | - Absolutely uncertain - Little certain - Somewhat certain - Mostly certain - Completely certain | Not at all sure      A bit sure      Quite sure      Very sure      Completely sure |
| 1. Wenn ich Schmerzen habe, ist es schrecklich und ich fühle mich überwältigt. | 1. When/If I have pain, it is dreadful, and I feel overwhelmed. | 1. When I have pain it is terrible and I feel helpless. |
| - Nie - Selten - Manchmal - Oft - Sehr oft | - Never - Seldom - Sometimes - Often - Very often | Never      Seldom      Sometimes      Often      Very often |
| 1. Haben Sie sich niedergeschlagen gefühlt? | 1. Have you felt depressed/ downtrodden? | 1. Have you ever felt depressed? |
| - Nie - Selten - Manchmal - Oft - Sehr oft | - Never - Seldom - Sometimes - Often - Very often | Never      Seldom      Sometimes      Often  Very often |
| 1. Haben Sie in Bezug auf Ihre Wirbelsäule Angst um Ihre Gesundheit? | 1. With respect to your spine, do you fear for your health? | 1. With regard to your spine, have you had fears about your health? |
| - Nie - Selten - Manchmal - Oft - Sehr oft | - Never - Seldom - Sometimes - Often - Very often | Never      Seldom      Sometimes      Often      Very often |
| 1. Beeinflusst Ihre Wirbelsäule Ihre Konzentrationsfähigkeit bei Unterhaltungen, beim Lesen und beim Fernsehen? | 1. Does your spine impact your ability to concentrate while conversing, reading, and watching television? | 1. Does your spine affect your ability to concentrate during conversation, while reading or watching television? |
| - Nie - Selten - Manchmal - Oft - Sehr oft | - Never - Seldom - Sometimes - Often - Very often | Never      Seldom      Sometimes      Often      Very often |
| 1. Haben Sie das Gefühl, dass Ihre persönlichen Beziehungen aufgrund des Zustandes Ihrer Wirbelsäule beeinflusst werden? | 1. Do you have the feeling that the state of your spine is impacting your personal relationships? | 1. Do you have the feeling that your personal relationships are influenced due to your spine condition? |
| - Nie - Selten - Manchmal - Oft - Sehr oft | - Never - Seldom - Sometimes - Often - Very often | Never      Seldom      Sometimes      Often      Very often |
| 1. Fühlen Sie sich wohl, wenn Sie neue Menschen kennenlernen? | 1. Do you feel comfortable meeting new people? | 1. Do you feel good when you meet new people? |
| - Nie - Selten - Manchmal - Oft - Sehr oft | - Never - Seldom - Sometimes - Often - Very often | Never      Seldom      Sometimes      Often      Very often |
| 1. Sind Sie mit den Behandlungsergebnissen Ihres Wirbelsäulentumors zufrieden? | 1. Are you satisfied with the results of your spine tumor treatment? | 1. Are you satisfied with the treatment of your spine tumor? |
| - Sehr zufrieden - Etwas zufrieden - Weder zufrieden noch unzufrieden - Etwas unzufrieden - Sehr unzufrieden | - Very satisfied - Somewhat satisfied - Neither satisfied nor unsatisfied - Somewhat unsatisfied - Very unsatisfied | Very satisfied      Somewhat satisfied      Neither satisfied nor unsatisfied      Somewhat unsatisfied      Very unsatisfied |
| 1. Würden Sie dieselbe Wirbelsäulentumorbehandlung wieder wählen? | 1. Would you pick the same spine tumor treatment again? | 1. Would you choose the same spine tumor treatment again? |
| - Sicher ja - Wahrscheinlich ja - Nicht sicher - Wahrscheinlich nicht - Sicher nicht | - Definitely yes - Probably yes - Unsure - Probably not - Definitely not | Surely yes      Probably yes      Not sure      Probably not      Definitely not |
| 1. Wie hat sich die Behandlung Ihrer Wirbelsäule auf Ihre körperlichen Fähigkeiten und Ihre Möglichkeiten, den Aktivitäten des täglichen Lebens nachzugehen, ausgewirkt? | 1. How has the treatment of your spine affected your physical abilities and your possibilities to accomplish the activities of daily life? | 1. How did the treatment of your spine affect your physical capabilities and your ability to complete your daily activities? |
| - Sehr verbessert - Etwas verbessert - Keine Veränderung - Etwas verschlechtert - Sehr verschlechtert | - Very improved - Somewhat improved - No change - Somewhat deteriorated - Very deteriorated | very much improved      Somewhat improved      No change      Somewhat worse      Much worse |
| 1. Wie hat die Behandlung ihrer Wirbelsäule die Funktionen ihres Rückenmarks und / oder Ihre Nervenfunktion beeinflusst? | 1. How has the treatment of your spine impacted the function of your spinal fluid and / or your nerve function? | 1. How did the treatment of your spine influence the function of your spinal cord/ or nerves? |
| - Sehr verbessert - Etwas verbessert - Keine Veränderung - Etwas verschlechtert - Sehr verschlechtert | - Much improved - Somewhat improved - No change - Somewhat deteriorated - Very deteriorated | Very much improved      Somewhat improved      No change      Somewhat worse  Much worse |
| 1. Wie hat Ihre Behandlung den Wirbelsäulenschmerz insgesamt beeinflusst? | 1. How has the treatment impacted your spine pain altogether? | 1. How did the treatment of your spine influence your pain in general? |
| - Sehr verbessert - Etwas verbessert - Keine Veränderung - Etwas verschlechtert - Sehr verschlechtert | - Much improved - Somewhat improved - No change - Somewhat deteriorated - Very deteriorated | Very much improved      Somewhat improved      No change      Somewhat worse      Much worse |
| 1. Wie hat die Behandlung Ihrer Wirbelsäule Ihre Depression/Niedergeschlagenheit und Angst beeinflusst? | 1. How has the treatment of your spine impacted your depression/ downtroddenness and fears? | 1. How did the treatment of your spine influence your depression and fear? |
| - Sehr verbessert - Etwas verbessert - Keine Veränderung - Etwas verschlechtert - Sehr verschlechtert | - Much improved - Somewhat improved - No change - Somewhat deteriorated - Very deteriorated | Very much improved      Somewhat improved      No change      Somewhat worse      Much worse |
| 1. Wie hat die Behandlung Ihrer Wirbelsäule Ihre gesellschaftlichen Fähigkeiten beeinflusst? | 1. How has the treatment of your spine impacted your social skills? | 1. How did the treatment of your spine influence your social capabilities? |
| - Sehr verbessert - Etwas verbessert - Keine Veränderung - Etwas verschlechtert - Sehr verschlechtert | - Much improved - Somewhat improved - No change - Somewhat deteriorated - Very deteriorated | Very much improved      Somewhat improved      No change      Somewhat worse      Much worse |
