# Supplementary material for: eCross-cultural adaptation of the spine oncology-specific SOSGOQ2.0 questionnaire to German language and the assessment of its validity and reliability in the clinical setting
Source: BMC Cancer. 2021 Sep 23;21:1044. doi: 10.1186/s12885-021-08578-x (PMC8459467; doi:10.1186/s12885-021-08578-x)
Supplement: Supplementary file 2 — Additional file 2: Table A2 - Adaptation of question 20 into German language. Note that the answer categories remain unchanged, however, the scale must be reversed. [file 12885_2021_8578_MOESM2_ESM.docx]

| **No. Question** | **Original German wording** | **New adaptation proposed here** | |
| --- | --- | --- | --- |
|  |  |  |  |
| 20 | Fühlen Sie sich wohl, wenn Sie neue Menschen kennenlernen? | Fühlen Sie sich aufgrund Ihrer Wirbelsäulenerkrankung unwohl, wenn Sie neue Menschen kennenlernen? | |
|  |  |  |  |
|  |  |  |  |
|  |  |  |  |
