# Supplementary material for: eCross-cultural adaptation of the spine oncology-specific SOSGOQ2.0 questionnaire to German language and the assessment of its validity and reliability in the clinical setting
Source: BMC Cancer. 2021 Sep 23;21:1044. doi: 10.1186/s12885-021-08578-x (PMC8459467; doi:10.1186/s12885-021-08578-x)
Supplement: Supplementary file 3 — Additional file 3: A3 - Supporting Information - Scoring manual SOSGOQ2.0_GER (German version) [file 12885_2021_8578_MOESM3_ESM.pdf]

A3 Supporting Information - Scoring manual SOSGOQ2.0_GER (Version in deutscher Sprache)

**Domäne**	**Fragen (fortlaufend nummeriert)**
Physische Funktionsfähigkeit	1*, 2*, 3*, 4*, 5*, 6
Schmerzen	11*, 12*, 13*, 14, 15*
Psychische Gesundheit	16*, 17*
Soziale Funktionsfähigkeit	18*, 19*, 20
**Einzelfragen zur Neurologischen Funktionsfähigkeit (zur quantitativen Bewertung des allgemeinen Gesundheitszustandes nicht relevant)**	
Beine	7*
Arme	8*
Blasenfunktion	10*
Darmfunktion	9*

Hinweis: * Die Fragenbewertung muss für die Berechnung umgekehrt werden.
(z.B. '1' → '5', '2' → '4', '4' → '2', '5' → '1')

Berechnung der Punktzahl pro Domäne

Die Umwandlung der Punktzahl pro Domäne in eine Skala von 0-100 wird mit Hilfe des folgenden Algorithmus durchgeführt:

$$\frac{(roher\ Punktwert - niedrigste\ mögliche\ rohe\ Punktzahl)}{(höchstmögliche\ rohe\ Punktzahl - niedrigste\ mögliche\ rohe\ Punktzahl)} \times 100$$

Roher Punktwert = Summe der Einzelfragen innerhalb einer Domäne

Eine Punktwert wird berechnet, wenn mindestens 50% der Fragen einer Domäne beantwortet sind (oder die Hälfte plus eins im Falle einer ungeraden Anzahl von Fragen)[#]. Wenn mehr als 50% der Fragen der Domäne fehlen, sollte der Punktwert für diese Domäne nicht berechnet werden. Eine höhere Punktzahl entspricht einer höheren Funktionsfähigkeit im Bereich der

körperlichen und sozialen Funktion. Eine höhere Punktzahl korrespondiert mit einem niedrigeren Symptomniveau im Bereich Schmerz und psychische Gesundheit.

unbekannter Grad der Übereinstimmung zwischen partiellen und vollständigen Antwortdatensätzen.

Einzelfragen zur neurologischen Funktionsfähigkeit

Hierbei handelt es sich um Fragen zu Einzelsymptomen (Fragen 7-10). Ein Punktwert kann berechnet werden, um die Berichterstattung in Studien zu erleichtern; zur Interpretation des Punktwertes sollten die einzelnen Fragen jedoch separat inspiziert werden, um den Ort der neurologischen Beeinträchtigung zu bestimmen. Die Umwandlung in einen Punktwert von 0-100 kann mit Hilfe des folgenden Algorithmus durchgeführt werden:

$$\frac{(Antwortkategorie - \ niedrigste\ mögliche\ Antwortkategorie)}{(höchstmögliche\ Antwortkategorie - \ niedrigste\ mögliche\ Antwortkategorie)} \times 100$$

Niedrigste mögliche Antwortkategorie = 1, höchstmögliche Antwortkategorie = 5

Eine 25-Punkte-Änderung in der Bewertung spiegelt dann eine Änderung um einen Punkt in der Antwortkategorie wider.

Gesamtpunktzahl für den SOSGOQ2.0 _GER

Eine Gesamtpunktzahl für den SOSGOQ2.0_GER kann unter Verwendung der Domänen physische Funktion, Schmerz, psychische Gesundheit und soziale Funktion berechnet werden.

$$\frac{Physische\ Funktionalität + Schmerzen + psychische\ Gesundheit + soziale\ Funktionalität}{4}$$

Wenn für eine der Domänen der Punktwert aufgrund fehlender Beantwortung nicht berechnet werden kann, kann folglich die Gesamtpunktzahl auch nicht berechnet werden.

Post-Therapie-Fragen

Bei den Post-Therapie-Fragen handelt es sich um Einzelfragen (Fragen 21-27). Die Umwandlung in eine Punktzahl von 0-100 wird mit Hilfe des folgenden Algorithmus durchgeführt:

$$\frac{(Antwortkategorie - \ niedrigste\ mögliche\ Antwortkategorie)}{(höchstmögliche\ Antwortkategorie - \ niedrigste\ mögliche\ Antwortkategorie)} \times 100$$

Niedrigste mögliche Antwortkategorie = 1, höchstmögliche Antwortkategorie = 5

Eine 25-Punkte-Änderung in der Bewertung spiegelt dann eine Änderung um einen Punkt in der Antwortkategorie wider. Eine höhere Punktzahl korrespondiert mit einem höheren Grad an Zufriedenheit und Verbesserung nach der Therapie. Die Verwendung der Post-Therapie-Fragen wird während der Nachsorge zusammen mit den Hauptfragen (1-20) empfohlen, um die verschiedenen Aspekte der Lebensqualität zu bewerten.
