# Supplementary material for: eCross-cultural adaptation of the spine oncology-specific SOSGOQ2.0 questionnaire to German language and the assessment of its validity and reliability in the clinical setting
Source: BMC Cancer. 2021 Sep 23;21:1044. doi: 10.1186/s12885-021-08578-x (PMC8459467; doi:10.1186/s12885-021-08578-x)
Supplement: Supplementary file 4 — Additional file 4: Structure of the Spine Oncology Study Group Outcomes Questionnaire 2.0 GERMAN (SOSGOQ2.0_GER) culturally adapted to German speaking people. Questions 1–6 represent the domain Physical Functioning, 7–10 Neurological Functioning, 11–15 Pain, 16/17 Mental Health, 18–20 Social Functioning, and 21–27 are post-therapy questions. In the supplement you will find a comparison of the back translated questions into English language performed independently by two native speakers and the final German adaptions (Table A1) as well as a German scoring manual (Table A3). [file 12885_2021_8578_MOESM4_ESM.docx]

**Hinweise:** In diesem Fragebogen werden Sie gebeten, Ihren Gesundheitszustand einzuschätzen. Bitte betrachten Sie beim Beantworten der Fragen Ihre Fähigkeiten und Symptome innerhalb der letzten 4 Wochen. Es ist wichtig, dass Sie jede Frage SELBST beantworten. Bitte geben Sie pro Frage NUR EINE ANTWORT an. Die Fragen 21-27 sollten nur NACH abgeschlossener Behandlung zu den Nachuntersuchungen beantwortet werden.

Patientenname:________________________________

Datum (DD/MM/JJJJ):___________________________

Patientennummer:______________________________

(vom medizinischen Fachpersonal auszufüllen)

**Vom PATIENTEN auszufüllen**

1. **Wie beurteilen Sie Ihre Aktivität?**

- Alle Aktivitäten ohne Einschränkung
- Moderate Aktivitäten außer Haus
- Mobilität auf zu Hause beschränkt
- Mobilität vom Bett zum Stuhl
- Bettgebunden

1. **Wie beurteilen Sie Ihre Arbeitsfähigkeit
   (Beruf/Haushalt)?**

- Uneingeschränkt
- 4-8 Stunden am Tag
- 2-4 Stunden am Tag
- Weniger als 2 Stunden am Tag
- Keine Arbeit möglich

1. **Sind Sie aufgrund Ihrer Wirbelsäule in Ihrer Fähigkeit für sich selbst zu sorgen eingeschränkt?**

- Überhaupt nicht
- Ein wenig
- Etwas
- Ziemlich
- Sehr stark

1. **Benötigen Sie Hilfe von anderen um sich außerhalb Ihres Zuhauses fortzubewegen?**

- Nie
- Selten
- Manchmal
- Oft
- Sehr oft

1. **Welche Unterstützung benötigen Sie beim
   Gehen?**

- Keine
- Eine Gehhilfe
- Einen Rollator / 2 Gehhilfen
- Hilfe durch andere
- Kein Gehen möglich

1. **Verlassen Sie das Haus für gesellschaftliche
   Aktivitäten?**

- Nie
- Selten
- Manchmal
- Oft
- Sehr oft

1. **Haben Sie eine Schwäche der Beine?**

- Keine
- Gelegentlich leicht
- Andauernd leicht
- Andauernd mäßig
- Andauernd stark

1. **Haben Sie eine Schwäche der Arme?**

- Keine
- Gelegentlich leicht
- Andauernd leicht
- Andauernd mäßig
- Andauernd stark

1. **Haben Sie Schwierigkeiten, Ihre Darmfunktion zu kontrollieren (außer bei Durchfall/Verstopfung)?**

- Nie
- Selten
- Manchmal
- Oft
- Sehr oft

1. **Haben Sie Schwierigkeiten, Ihre Blasenfunktion
   zu kontrollieren?**

- Nie
- Selten
- Manchmal
- Oft
- Katheter erforderlich

1. **In welchem Ausmaß haben Sie insgesamt
   Rücken-/Nackenschmerzen?**

- Keine
- Sehr leichte
- Leichte
- Mäßige
- Starke

1. **Wenn Sie sich in Ihrer bequemsten Körper-position befinden, haben Sie weiterhin Rücken-/ Nackenschmerzen (welche Ihren Schlaf einschränken)?**

- Nie
- Selten
- Manchmal
- Oft
- Sehr oft

1. **Wie oft beeinträchtigen Schmerzen Ihre
   Beweglichkeit (Sitzen, Stehen, Gehen)?**

- Nie
- Selten
- Manchmal
- Oft
- Dauernd

1. **Wie sicher fühlen Sie sich in Ihren Möglichkeiten, Ihren Schmerz selbständig zu bewältigen?**

- Überhaupt nicht sicher
- Wenig sicher
- Mäßig sicher
- Meistens sicher
- Völlig sicher

1. **Wenn ich Schmerzen habe, ist es schrecklich und ich fühle mich überwältigt.**

- Nie
- Selten
- Manchmal
- Oft
- Sehr oft

1. **Haben Sie sich niedergeschlagen gefühlt?**

- Nie
- Selten
- Manchmal
- Oft
- Sehr oft

1. **Haben Sie in Bezug auf Ihre Wirbelsäule Angst
   um Ihre Gesundheit?**

- Nie
- Selten
- Manchmal
- Oft
- Sehr oft

1. **Beeinflusst Ihre Wirbelsäule Ihre Konzentrationsfähigkeit bei Unterhaltungen, beim Lesen und
   beim Fernsehen?**

- Nie
- Selten
- Manchmal
- Oft
- Sehr oft

1. **Haben Sie das Gefühl, dass Ihre persönlichen
   Beziehungen aufgrund des Zustandes Ihrer
   Wirbelsäule beeinflusst werden?**

- Nie
- Selten
- Manchmal
- Oft
- Sehr oft

1. **Fühlen Sie sich wohl, wenn Sie neue Menschen kennenlernen?**

- Nie
- Selten
- Manchmal
- Oft
- Sehr oft

1. **Sind Sie mit den Behandlungsergebnissen Ihres Wirbelsäulentumors zufrieden?**

- Sehr zufrieden
- Etwas zufrieden
- Weder zufrieden noch unzufrieden
- Etwas unzufrieden
- Sehr unzufrieden

1. **Würden Sie dieselbe Wirbelsäulentumorbehandlung wieder wählen?**

- Sicher ja
- Wahrscheinlich ja
- Nicht sicher
- Wahrscheinlich nicht
- Sicher nicht

1. **Wie hat sich die Behandlung Ihrer Wirbelsäule auf Ihre körperlichen Fähigkeiten und Ihre Möglichkeiten, den Aktivitäten des täglichen Lebens nachzugehen, ausgewirkt?**

- Sehr verbessert
- Etwas verbessert
- Keine Veränderung
- Etwas verschlechtert
- Sehr verschlechtert

1. **Wie hat die Behandlung Ihrer Wirbelsäule die Funktionen Ihres Rückenmarks und/oder Ihre Nervenfunktion beeinflusst?**

- Sehr verbessert
- Etwas verbessert
- Keine Veränderung
- Etwas verschlechtert
- Sehr verschlechtert

1. **Wie hat Ihre Behandlung den Wirbelsäulenschmerz insgesamt beeinflusst?**

- Sehr verbessert
- Etwas verbessert
- Keine Veränderung
- Etwas verschlechtert
- Sehr verschlechtert

1. **Wie hat die Behandlung Ihrer Wirbelsäule Ihre
   Niedergeschlagenheit und Ängste beeinflusst?**

- Sehr verbessert
- Etwas verbessert
- Keine Veränderung
- Etwas verschlechtert
- Sehr verschlechtert

1. **Wie hat die Behandlung Ihrer Wirbelsäule Ihre
   gesellschaftlichen Aktivitäten beeinflusst?**

- Sehr verbessert
- Etwas verbessert
- Keine Veränderung
- Etwas verschlechtert
- Sehr verschlechtert
